# Phase 1b study of berzosertib and cisplatin in patients with advanced triple-negative breast cancer

**DOI:** 10.1038/s41523-022-00406-0

**Published:** 2022-04-07

**Authors:** Melinda L. Telli, Sara M. Tolaney, Geoffrey I. Shapiro, Mark Middleton, Simon R. Lord, Hendrik Tobias Arkenau, Andrew Tutt, Vandana Abramson, Emma Dean, Tufia C. Haddad, Robert Wesolowski, Jordi Ferrer-Playan, Thomas Goddemeier, Thomas Grombacher, Jennifer Dong, Patricia Fleuranceau-Morel, Ivan Diaz-Padilla, Ruth Plummer

**Affiliations:** 1 Stanford University School of Medicine, Stanford, CA USA; 2 Dana-Farber Cancer Institute and Harvard Medical School, Boston, MA USA; 3 University of Oxford, Oxford, UK; 4 Sarah Cannon Research Institute, HCA Healthcare, London, UK; 5 University College London, London, UK; 6 Breast Cancer Now Toby Robins Research Centre, The Institute of Cancer Research and Kings College, London, UK; 7 Guy’s and St Thomas’ NHS Foundation Trust, London, UK; 8 Vanderbilt University Medical Center, Vanderbilt-Ingram Cancer Center, Nashville, TN USA; 9 The University of Manchester and The Christie NHS Foundation Trust, Manchester, UK; 10 Mayo Clinic, Rochester, MN USA; 11 The Ohio State University Comprehensive Cancer Center, Columbus, OH USA; 12 Ares Trading SA, Eysins, Switzerland, an affiliate of Merck KGaA, Darmstadt, Germany; 13 the healthcare business of Merck KGaA, Darmstadt, Germany; 14 EMD Serono, Billerica, MA USA; 15 Newcastle University and Northern Centre for Cancer Care, Newcastle Hospitals NHS Foundation Trust, Newcastle upon Tyne, UK; 16 Present Address: Oncology R&D, AstraZeneca, Cambridge and Alderley Park, Macclesfield, UK; 17 Present Address: GlaxoSmithKline, Zug, Switzerland

**Keywords:** Breast cancer, Cancer therapy

## Abstract

Platinum derivatives are commonly used for the treatment of patients with metastatic triple-negative breast cancer (TNBC). However, resistance often develops, leading to treatment failure. This expansion cohort (part C2) of the previously reported phase 1b trial (NCT02157792) is based on the recommended phase 2 dose of the combination of the ataxia-telangiectasia and Rad3-related (ATR) inhibitor berzosertib and cisplatin observed in patients with advanced solid tumors, including TNBC. Forty-seven patients aged ≥18 years with advanced TNBC received cisplatin (75 mg/m^2^; day 1) and berzosertib (140 mg/m^2^; days 2 and 9), in 21-day cycles. Berzosertib was well tolerated, with a similar toxicity profile to that reported previously for this combination. The overall response rate (90% confidence interval) was 23.4% (13.7, 35.8). No relevant associations were observed between response and gene alterations. Further studies combining ATR inhibitors with platinum compounds may be warranted in highly selected patient populations.

## Introduction

Triple-negative breast cancer (TNBC) accounts for ~10–20% of all patients with breast cancer and is traditionally defined by the lack of expression of the estrogen and progesterone receptors, alongside no overexpression or amplification of the human epidermal growth factor receptor 2 (HER2)^1^. TNBC has the poorest prognosis amongst all breast cancer subtypes^2^; the reported 5-year survival for patients with any metastatic breast cancer is ~27%, compared with 11% in patients with metastatic TNBC^3,4^. At least four different subtypes of breast cancers have been identified according to their gene expression profile, namely: luminal A, luminal B, HER2-enriched, and basal-like (basal)^5,6^.

In the setting of advanced TNBC, carboplatin has previously demonstrated similar efficacy and a more favorable toxicity profile compared to docetaxel^7–9^, and therefore platinum derivates have commonly been used for the treatment of metastatic disease^10^. However, the treatment options for patients with advanced TNBC have expanded in the last few years as new therapeutic modalities, such as immune checkpoint inhibitors and antibody–drug conjugates, have become available^11–13^. Poly (ADP-Ribose) Polymerase (PARP) inhibitors have also been approved for use in patients with germline Breast Cancer Gene (*BRCA) 1/2-*mutated, HER2-negative metastatic breast cancer. This includes patients with TNBC who have germline *BRCA1/2* mutations, with clinical benefits also extending to those with somatic *BRCA1/2* mutations, although it is unclear whether similar benefits will be observed in patients with TNBC^14,15^ without germline or somatic *BRCA1/2* mutations. Despite some progress being made, there remains a significant unmet medical need to improve treatment outcomes for patients with advanced TNBC.

Ataxia telangiectasia and Rad3-related (ATR) and ataxia-telangiectasia-mutated (ATM) protein kinases are members of the DNA damage response (DDR) family of proteins. Both ATM and ATR are key regulators of cell cycle checkpoint control and predominantly direct repair of DNA via homologous recombination^16^. Loss or inactivation of a single member of the DDR family of proteins has been associated with a greater reliance on other family members to respond to DNA damage, including damage induced by platinum-based chemotherapy. Preclinical experiments conducted using human ovarian and breast cancer cell lines suggest that their sensitivity to DNA-damaging agents may be increased when used in combination with ATR inhibitors^17^. Preclinical evidence also suggests that tumor protein 53 (*TP53*) mutant status correlates with the response to ATR inhibition in combination with DNA damaging agents^18–20^. Since basal breast cancers often present with a high frequency of *TP53* mutations^21,22^, combining ATR inhibitors and DNA-damaging chemotherapy is a theoretically efficacious therapeutic approach in this setting.

Berzosertib (formerly M6620, VX-970) is an intravenously (i.v.) administered, highly potent and selective, first-in-class inhibitor of ATR^23^. Preclinical studies have demonstrated the ability of ATR inhibitors to sensitize breast cancer cell lines to platinum-based chemotherapy, namely cisplatin^17^. The part A and B cohorts of the first-in-human study (NCT02157792) phase 1 trial of berzosertib established the recommended phase 2 dose (RP2D) and demonstrated its tolerability, both as a single agent and in combination with certain chemotherapies; preliminary signs of efficacy were also observed in patients with a range of solid tumor types, including TNBC^24,25^. The tolerability, preliminary signs of efficacy, and favorable pharmacokinetics (PK) identified in this study have been confirmed in other clinical studies of berzosertib^26–28^. A berzosertib–topotecan combination has also been evaluated in a recent proof-in-concept phase 2 study, reporting an objective response rate of 36% (9/25) and a median duration of response of 6.4 months in patients with SCLC, including platinum-resistant patients^29^. Parts A and B of NCT02157792 were dose escalation studies that established the RP2D of berzosertib in combination with gemcitabine (part A; berzosertib 210 mg/m^2^ [days 2 and 9] in combination with gemcitabine 1000 mg/m^2^ [days 1 and 8])^24^ and cisplatin (part B; berzosertib 140 mg/m^2^ [days 2 and 9] in combination with cisplatin 75 mg/m^2^ [day 1])^25^ in 21-day cycles.

Here we report the results from the C2 expansion cohort of the same study, which investigated the safety and tolerability, efficacy, PK, and potential predictive biomarkers of berzosertib in combination with cisplatin in patients with advanced TNBC whose tumors were germline *BRCA1/2* wild-type and basal subtype.

## Results

### Patient demographics and disposition

Forty-seven patients were enrolled into this study, all of whom were included in the safety analysis set (SAF) and modified full analysis set (mFAS) for efficacy (Table 1). The PK analysis set (PAS) included all enrolled patients who received at least one dose of berzosertib and provided at least one measurable post-dose concentration; 41 patients were included in the PAS, with 6 patients excluded as they did not have a measurable post-dose PK berzosertib concentration. The modified primary efficacy set (mPES) included all patients in the mFAS who were basal subtype and *BRCA1/2* germline wild-type; 35 patients were included in the mPES, with 12 patients excluded due to a lack of BRCA1/2 gWT status data or were not basal subtype. Among all patients, 34 (72.3%) were *TP53* mutant; 2 (4.3%) were *TP53* wild-type; 31 (66%) were *BRCA1/2* germline wild-type; and 36 (76.6%) were basal subtype, based on Prediction Analysis of Microarray 50 (PAM50). In the 7 patients who were not basal subtype, 4 (57.1%) had a *TP53* mutation, 1 (14.3%) was *TP53* wild-type and 2 (28.6%) had an unknown *TP53* mutational status; in the 36 (76.6%) patients who were basal subtype, 29 (81%) had a *TP53* mutation, 1 (3%) was *TP53* wild-type, and 6 (17%) had an unknown *TP53* mutational status. Four patients had an unknown subtype (Table 2).

**Table 1:** Patient demographics and baseline characteristics.

Characteristic, *N* (%) unless stated	Total *N* = 47
Sex	
Male	0
Female	47 (100)
Ethnicity	
Caucasian/White	41 (87.2)
Non-Caucasian/non-White	3 (6.4)
Age, years; median (range)	48.0 (35–73)
Baseline ECOG PS	
0	25 (53.2)
1	22 (46.8)
Prior anticancer therapy	
Chemotherapy	45 (95.7)
Immunotherapy	1 (2.1)
Other	11 (23.4)
Missing	2 (4.3)
Number of previous anticancer therapy regimens	
Neoadjuvant	16 (34.0)
Adjuvant	27 (57.4)
1^st^ line, metastatic disease	24 (51.1)
2^nd^ line, metastatic disease	10 (21.3)
>2^nd^ line, metastatic disease	5 (10.6)
Missing	2 (4.3)
*TP53*^a^	
Wild type	2 (4.3)
Mutant	34 (72.3)
Missing	11 (23.4)
*BRCA1/2*^b^	
Wild type	31 (66)
Mutant	5 (10.6)
Missing	11 (23.4)
Basal subtype^c^	
Yes	36 (76.6)
No	7 (14.9)
Missing	4 (8.5)

*ATM* ataxia-telangiectasia mutated, *BRCA1/2* breast cancer gene 1/2, *ECOG PS* Eastern Cooperative Oncology Group Performance Status, *TP53* tumor protein 53.

^a^Only patients with biomarker status determined by FoundationOne® CDx next generation sequencing were reported.

^b^Patients with unknown *BRCA1/2* status were prospectively tested at screening by BRCAnalysis assay (Myriad Genetics); patients that were found to be *BRCA1/2* germline mutant were still enrolled in the study.

^c^Assessment for basal subtype was performed retrospectively via PAM50 analysis (Prosigna).

**Table 2:** *TP53* mutational status.

*TP53* status	Basal subtype (*N* = 36)	Non-basal subtype (*N* = 7)
Wild type	1 (3.0)	1 (14.3)
Mutant	29 (81.0)	4 (57.1)
Missing	6 (17.0)	2 (28.6)

*TP53* tumor protein 53.

In all, 24 (51.1%) patients had previously received only one line of therapy for metastatic disease, 10 (21.3%) patients had received two lines of therapy, and 5 (10.6%) received more than two lines of therapy for metastatic disease.

All 47 patients in the mFAS received at least one dose of berzosertib, 46 (97.9%) received at least one dose of cisplatin, and 12 (25.5%) received at least one dose of carboplatin; the patient who received berzosertib without cisplatin received at least one dose of carboplatin. Thirty-eight (80.9%) patients completed treatment with berzosertib, while nine (19.1%) discontinued treatment, primarily due to patient decision (four, 8.5%) or adverse events (AEs; two, 4.3%). Sixteen patients (34%) discontinued cisplatin, primarily due to AEs (12.8%) and remained on berzosertib monotherapy until PD. The median (range) duration of treatment for berzosertib in combination with cisplatin was 15.0 (5 cycles) (2.0, 137.1) weeks. No patients died during this study.

### Efficacy

**Fig. 1:**
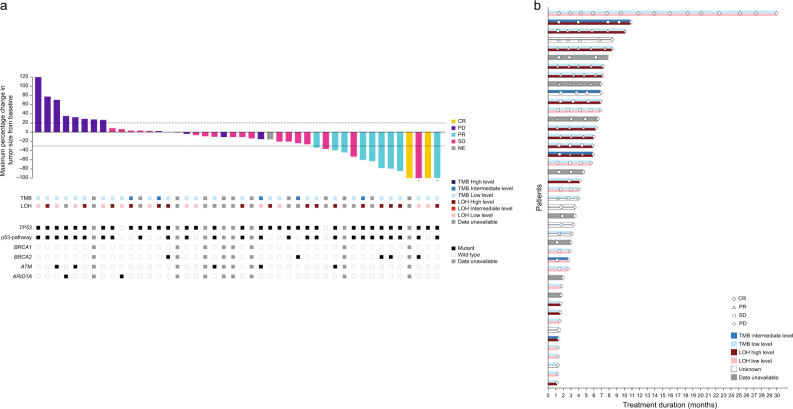
Best percentage change in tumor size from baseline and tumor response by LOH and TMB scores (modified full analysis set). **a** Best percentage change in tumor size from baseline, **b** tumor response by LOH and TMB scores. Patients without on-treatment target lesion measurements are not shown in Fig. 1a. Patients without response assessments are not shown in Fig. 1b. All response categories were determined according to RECIST 1.1 criteria. Full details of mutations can be found in Supplementary Table 5. CR complete response, LOH loss of heterozygosity, PD progressive disease, PR partial response, RECIST Response Evaluation Criteria in Solid Tumors, SD stable disease, TMB tumor mutational burden.

Forty-seven patients were included in the mFAS; however four patients were non-evaluable. The objective response rate (ORR [90% confidence interval (CI)]) was 11/47 (23.4%) patients (13.7, 35.8), the best overall response (BOR) was complete response (CR) for two (4.3%) patients, partial response (PR) for nine (19.1%) patients, and stable disease (SD) for 18 (38.3%) patients (Table 3 and Fig. 1a); all responses were confirmed. For patients with SD (*N* = 18), 3 patients (16.7%) progressed within 3 months, 10 (55.6%) patients progressed within 3–6 months, and 5 (27.8%) patients progressed within 6–12 months. BOR was also retrospectively stratified by prior lines of treatment for metastatic disease (Supplementary Table 1); all patients who achieved a BOR of CR were previously treated only in the neoadjuvant/adjuvant setting, while no patients who had received more than two lines of therapy for metastatic disease achieved a response.

**Table 3:** Efficacy responses (modified full analysis set, *N* = 47).

Efficacy outcome	Patients, *n* (%) unless stated
BOR	
CR	2 (4.3)
PR	9 (19.1)
SD	18 (38.3)
PD	14 (29.8)
Not evaluable	4 (8.5)
ORR, *N* (%), (90% CI)	11 (23.4) (13.7, 35.8)
DCR, *N* (%), (90% CI)	29 (61.7) (48.7, 73.6)
Median PFS (months), (90% CI)	4.0 (2.8, 6.0)
Median OS (months), (90% CI)	12.4 (7.8, 14.5)
Median DOR (months), (90% CI)	6.0 (5.1, nd)

DCR was defined as the proportion of patients with disease control, defined as a BOR of CR, PR, or SD.

*BOR* best overall response, *CI* confidence interval, *CR* complete response, *DCR* disease control rate, *DOR* duration of response, *nd* not defined, *ORR* overall response rate, *OS* overall survival, *PD* progressive disease, *PFS* progression-free survival, *PR* partial response, *SD* stable disease.

The median duration of response (DOR; [90% CI]) was 6.0 months (5.1, not defined). Median progression-free survival (PFS; [90% CI]) was 4.0 months (2.8, 6.0), and median overall survival (OS; [90% CI]) was 12.4 months (7.8, 14.5).

Similar results were observed for the mPES: the ORR (90% CI) was 9/35 (25.7%) patients (14.1, 40.6), 2 (5.7%) patients had a BOR of CR, 7 (20.0%) had a BOR of PR, and 15 (42.9%) had a BOR of SD; 10 (28.6%) patients had a BOR of PD. The ORR for patients who were germline *BRCA1/2* wild-type vs mutant were 7/31 (22.6%) patients vs 2/5 (40.0%) patients, respectively, while for patients who were basal subtype vs those who were not, the ORRs were 9/36 (25.0%) patients vs 1/7 (14.3%) patients, respectively. However, no formal statistical comparison for these subgroups was made. Further information on ORR by biomarker status can be found in Table 4.

**Table 4:** ORR for selected biomarker subgroups; modified full analysis set (*N* = 47).

Gene	Patients, *n*	Responses, *n*	ORR, % (90% CI)
*TP53*^a^			
Wild type	2	0	0.0 (0.0, 77.6)
Mutant	34	9	26.5 (14.6, 41.6)
*ARID1A*			
Wild type	34	9	26.5 (14.6, 41.6)
Mutant	2	0	0.0 (0.0, 77.6)
*ATM*			
Wild type	31	8	25.8 (13.5, 41.8)
Mutant	5	1	20.0 (1.0, 65.7)
Germline *BRCA1/2*			
Wild type	31	7	22.6 (11.1, 38.3)
Mutant	5	2	40.0 (7.6, 81.1)
Basal subtype^b^			
Yes	36	9	25.0 (13.7, 39.6)

*ARID1A* AT-rich interaction domain 1A, *ATM* ataxia-telangiectasia-mutated, *BRCA1/2* breast cancer gene 1/2, *CI* confidence interval, *ORR* overall response rate, *PAM50* Prediction Analysis of Microarray 50, *TP53* tumor protein 53.

^a^Only patients with biomarker status determined by FoundationOne® CDx next-generation sequencing were reported.

^b^Basal subtype was determined via PAM50 analysis (Prosigna) and was performed retrospectively.

### Safety and tolerability

A summary of treatment-emergent AEs (TEAEs) is presented in Table 5. The most common TEAEs, which occurred in more than 50% of patients, were nausea (*N* = 39, 83.0%), fatigue (*N* = 32, 68.1%), neutropenia (*N* = 29, 61.7%), and vomiting (*N* = 28, 59.6%); a full list of TEAEs affecting ≥20% of patients can be found in Table 5. TEAEs of grade ≥3 occurred in 32 patients (62.1%). TEAEs of grade ≥3 occurring in more than 10% of patients were neutropenia (*N* = 18, 38.3%), anemia (*N* = 12, 25.5%), thrombocytopenia (*N* = 6, 12.8%), and vomiting (*N* = 6, 12.8%). Febrile neutropenia occurred in two (4.3%) patients. Forty-five (95.7%) patients had a berzosertib-related TEAE; of these, 27 (57.4%) had a related TEAE of grade ≥3. There were no notable adverse events relating to laboratory results, vital signs or electrocardiogram (ECG) measurements.

**Table 5:** Overview of TEAEs for berzosertib and cisplatin (safety analysis set, *N* = 47).

Patients, *N* (%)	Berzosertib + Cisplatin Any grade	Berzosertib + Cisplatin Grade ≥3
	*N* = 47	*N* = 47
TEAE	47 (100)	36 (76.6)
Berzosertib-related TEAE	45 (95.7)	27 (57.4)
Cisplatin or carboplatin-related TEAE	47 (100)	31 (66.0)
Berzosertib or cisplatin or carboplatin-related TEAE	47 (100)	32 (68.1)
TEAEs occurring in ≥20% of patients		
Nausea	39 (83.0)	4 (8.5)
Fatigue	32 (68.1)	1 (2.1)
Neutropenia	29 (61.7)	18 (38.3)
Vomiting	28 (59.6)	6 (12.8)
Tinnitus	21 (44.7)	0
Anemia	19 (40.4)	12 (25.5)
Headache	18 (38.3)	0
Diarrhea	16 (34.0)	0
Constipation	14 (29.8)	0
Dizziness	11 (23.4)	0
Decreased appetite	10 (21.3)	0
Serious TEAE	15 (31.9)	13 (27.7)
Berzosertib-related serious TEAE	10 (21.3)	8 (17.0)
Cisplatin or carboplatin-related serious TEAE	10 (21.3)	8 (17.0)
Berzosertib or cisplatin or carboplatin-related serious TEAE	10 (21.3)	8 (17.0)
TEAEs leading to permanent discontinuation of treatment		
TEAE leading to permanent discontinuation of berzosertib	5 (10.6)	NR
Berzosertib-related TEAE leading to permanent discontinuation of berzosertib	3 (6.4)	NR
Cisplatin-related TEAE leading to permanent discontinuation of cisplatin	8 (17.0)	NR
Carboplatin-related TEAE leading to permanent discontinuation of carboplatin	3 (6.4)	NR
TEAE leading to a dose reduction in at least one study drug	11 (23.4)	NR
Berzosertib-related TEAE leading to dose reduction in berzosertib	3 (6.4)	NR
Cisplatin-related TEAE leading to dose reduction in cisplatin	8 (17.0)	NR
Carboplatin-related TEAE leading to dose reduction in carboplatin	1 (2.1)	NR
TEAE leading to temporary discontinuation of at least one study drug	24 (51.1)	NR
Berzosertib-related TEAE leading to temporary discontinuation of berzosertib	21 (44.7)	NR
Cisplatin-related TEAE leading to temporary discontinuation of cisplatin	15 (31.9)	NR
Carboplatin-related TEAE leading to temporary discontinuation of carboplatin	5 (10.6)	NR
TEAE leading to death	0	0

*NR* not reported, *TEAE* treatment-emergent adverse event.

In all, 15 (31.9%) patients had a serious TEAE, with 13 (27.7%) having a grade ≥3 serious TEAE. Of these patients, 10 (21.3%) reported a serious TEAE related to berzosertib, 8 (17%) related to cisplatin, and 2 (4.3%) related to carboplatin.

In total, 11 (23.4%) patients experienced a TEAE leading to a dose reduction in at least one study drug (3 [6.4%], 8 [17.0%], and 1 [2.1%] TEAEs leading to permanent dose reduction were related to berzosertib, cisplatin, and carboplatin, respectively).

In total, 24 (51.1%) patients experienced a TEAE leading to temporary discontinuation of at least one study drug (21 [44.7%], 15 [31.9%], and 5 [10.6%] TEAEs leading to temporary discontinuation were related to berzosertib, cisplatin, and carboplatin, respectively). Five (10.6%) patients in this expansion cohort permanently discontinued berzosertib during the study due to TEAEs, three of whom discontinued due to berzosertib-related TEAEs (one patient each experienced neutropenia [grade 2], anemia and thrombocytopenia [both grade 3], and peripheral neuropathy [grade 2]).

### Biomarkers

No conclusive association was identified between clinical outcome (ORR or PFS) and the mutational status of *TP53*, *AT-rich interaction domain 1A (ARID1A)*, *ATM* or *BRCA1/2* (Table 4 and Supplementary Table 2). Post-hoc exploratory biomarker assessments of loss of heterozygosity (LOH) status and tumor mutational burden (TMB) using Foundation Medicine’s (FMI) FoundationOne® CDx assay^30,31^ in these patients did not identify any statistically significant association between LOH and response or TMB and response (Fig. 1b). However, 7 of the 11 patients with a confirmed response had a high LOH score and 7 of the 16 total patients with a high LOH score responded to treatment, compared with only 1 of the 12 patients with a low LOH score. Although the association was not statistically significant (*p* = 0.09), the results suggest an enrichment of responders in tumors with high LOH (OR = 7.96) (Supplementary Table 3). No patients had a tumor with a high TMB score. In all, 7 of the 27 patients with a low TMB score had a confirmed response, compared with only 1 of the 5 patients with an intermediate TMB score.

A 40-year-old female patient with grade 3, metastatic, BRCA1/2 negative TNBC achieved a CR with a duration of 28.6 months, remaining in CR at the end of the study. The patient achieved a BOR of CR despite disease progression on previous chemotherapeutic treatment in the (neo)adjuvant setting. Further assessment of the biomarker status of this patient revealed heterozygous germline mutations in genes including *adenovirus E1A-associated cellular p300 transcriptional co-activator protein (EP300)*, *DNA polymerase delta 1, catalytic subunit (POLD1)*, and *Tumor Growth Factor Beta Receptor 2 (TGFBR2)*, all variants of unknown significance. Archival tumor demonstrated low LOH and TMB scores, somatic *TP53* mutation and several gene amplification events relating to *MYC* (copy number 18)*, CCNE1* (Cyclin E1; copy number 14), and *RAD21* (copy number 18).

### Pharmacokinetics

The PAS included 41 patients, with six patients excluded because their post-dose berzosertib concentration was outside the limit of quantification. The administered i.v. berzosertib dose of 140 mg/m^2^ was within the dose range that has previously shown dose-dependent berzosertib PK as monotherapy or in combination with either carboplatin or cisplatin^24–26^. The observed berzosertib concentration data in this expansion cohort were generally consistent with those reported previously at the same dose level^25^. Cisplatin had no apparent effect on berzosertib PK (Supplementary Fig. 1). Key berzosertib PK parameters from this study, presented as geometric mean (geometric coefficient of variance), were as follows: maximum observed concentration (*C*_max_ [ng/mL]) – 555 (42.9%); area under the curve (AUC) from start of infusion to the 4-h sampling time after start of infusion (AUC_0–4_ [ng.h/mL]) – 1110 (26.4%). The *C*_max_ in this expansion cohort was slightly lower than the 652 ng/mL (25%) and 854 ng/mL (63%) observed in the lead-in period and in the berzosertib and cisplatin combination cohort, respectively, at the same dose level in part B. The partial AUC_0–4_ was ~23% of the reported AUC_0–∞_ in part B^25^.

## Discussion

In this dose expansion cohort study, berzosertib 140 mg/m^2^ (days 2 and 9) administered alongside cisplatin 75 mg/m^2^ (day 1) in 21-day cycles was well tolerated in patients with advanced TNBC. The safety profile of this combination was broadly consistent with that of the individual agents and no new or unexpected safety signals were identified for berzosertib^26^. Although a high rate of treatment interruptions was observed with berzosertib, these did not translate into dose reductions or permanent treatment discontinuation. The rate of bone marrow toxicity observed in this study with a limited number of participants may indicate the potential of ATR inhibition to increase the hematologic toxicity of cisplatin and may be an on-target effect. However, these toxicities were generally manageable and did not result in significant dose reductions or treatment discontinuations.

The observed PK data were generally consistent with the PK data reported previously at the same dose levels^28^. A population PK model built based on pooled data from two phase 1 studies, including this expansion cohort, suggested that administration of cisplatin does not affect observed berzosertib PK parameters^28^. Although the effects of TNBC tumor type on clearance were estimated in the model, an association between tumor type and PK is not anticipated^28^.

With the implicit caveats associated with cross-trial comparisons, the response rate observed in this expansion cohort (ORR: 23.4%; DOR: 6.0 months) was broadly consistent with those observed in historical studies conducted in patients with advanced TNBC treated with platinum-based therapy (TNT trial, carboplatin ORR: 31.4%; TBCRC009 trial, ORR: 25.6%)^7,32^.

Although preclinical evidence suggested a potent synergistic effect of berzosertib and cisplatin on cancer cell survival, particularly in tumors with *TP53* mutations, this was not observed with the berzosertib and cisplatin doses utilized in this study^18–20^. The lower dose of berzosertib used in this study may have been insufficient for target engagement. For instance, a Phase 2 proof-of-concept study investigating a dose of 210 mg/m^2^ of berzosertib in combination with topotecan demonstrated durable tumor regression in patients with small-cell neuroendocrine cancers^29^, whereas no benefit in PFS was shown in a Phase 2 study investigating a dose of 90 mg/m^2^ berzosertib in combination with gemcitabine in patients with advanced urothelial carcinoma^33^. Additionally, patients in the clinical setting are likely to have tumors with complex genetic aberrations; hence, solely assessing *TP53* mutational status may not provide a robust assessment of p53 pathway impairment. Several co-occurring DDR alterations may be necessary to confer sensitivity to the combination of ATR inhibition and platinum-derived chemotherapeutics.

One patient with TNBC and several co-occurring DDR alterations experienced a CR lasting for 28.6 months. Further assessment of the biomarker status of this patient revealed a number of amplification events relating to *MYC*, *CCNE1*, and *RAD21*. This exceptional response might be explained in part by the presence of these specific gene amplifications. Overexpression of *MYC* and *CCNE1* (Cyclin E1) have been shown to increase replication stress, thus sensitizing tumors to ATR inhibition^34,35^. The role of *RAD21* is less clear in this context, as up-regulation of RAD21 has been shown to mitigate replication stress arising from *MYC* overexpression^36^. Future research may explain the complex interplay between co-occurring gene amplifications and ATR inhibition.

Taken together, no conclusive associations were observed for gene alterations linked to higher susceptibility to *ATR* inhibition or increased reliance on *ATR* in preclinical experiments, such as *ATM* and *ARID1A* alterations^37^. This may be due to the low patient numbers; for example, there were only five patients with an *ATM* mutation (Supplementary Table 4). However, an enrichment of responses would have been expected in patients with ATM loss, which raises questions related to the functionality of ATM, or how this is defined (i.e. based on loss-of-function mutation, mono-allelic vs bi-allelic loss, or protein expression). Other studies have pointed towards such an association between ATM protein loss and response to ATR inhibition^38^. The identification of biomarkers of response to ATR inhibitors continues to be a very active field of research.

As LOH can indicate the presence of historical homologous recombination deficiency^39,40^ and has also been reported to predict response to platinum-based neoadjuvant chemotherapy in patients with TNBC^41^, an exploratory biomarker analysis focused on evaluating genetic alterations and genomic signatures was conducted in this trial. Interestingly, there were a greater number of clinical responses in patients with a high LOH status (7/16) within this patient population compared with patients with a low LOH status (1/12).

In conclusion, the combination of berzosertib and cisplatin in patients with advanced, pretreated TNBC was well tolerated. Although the observed efficacy signal does not warrant further development of the present combination and dose in patients with TNBC in a phase 2/3 setting, the clinical evaluation of berzosertib in a very selective sub-population of patients with breast cancer might be of interest to confirm the role of certain genetic or molecular markers, such as LOH. Clinical trials evaluating berzosertib in combination with DNA damage-inducing chemotherapy, such as topotecan, are ongoing in a variety of solid tumors including small-cell lung cancer.

## Methods

### Study design

This was a multicenter, open-label, non-randomized, first-in-human, phase 1 study conducted in six parts (A, B, B2, C1, C2, C3; NCT02157792, registered 06 June 2014). Part C2 was a single-arm, dose expansion cohort evaluating the safety and preliminary efficacy of berzosertib combined with cisplatin in patients with advanced TNBC using the berzosertib RP2D identified in part B. Patients were enrolled across five sites in the UK and 12 in the USA, between 09 December 2015 (study initiation date) and 11 March 2020 (study completion date, when the final patient completed their last visit).

This study was conducted in accordance with the ethical principles of the International Council for Harmonisation guideline for Good Clinical Practice and the Declaration of Helsinki, as well as with applicable local regulations. The Clinical Study Protocol and all required associated documents were approved by the following responsible Institutional Review Boards (IRB) or Independent Ethics Committees of the individual study sites: The Washington University in St. Louis IRB, Vanderbilt University IRB, North East – Tyne & Wear South Research Ethics Committee, Northwestern University Biomedical IRB, Stanford University Administrative Panels on Human Subjects in Medical Research Board, Greenville Health System IRB, Mayo Clinic IRB, The University Hospitals IRB, US Oncology IRB, NRES Committee North East – Sunderland, Dana Farber Cancer Institute IRB, Western IRB. All patients were required to provide written informed consent prior to enrollment.

### Patients

Eligible patients were: ≥18 years of age; had advanced (locally advanced incurable or metastatic), histologically confirmed estrogen receptor, progesterone receptor, and HER2-negative breast cancer; adequate available historical tumor biopsies (core biopsy or surgical specimen); 0–2 prior therapies for the treatment of advanced breast cancer; and measurable disease according to Response Evaluation Criteria in Solid Tumors (RECIST) 1.1^42^. A minimum of 30 patients who were germline *BRCA1/2* wild-type and had a basal subtype, as assessed by PAM50, were to be enrolled into this study. If one or more of these characteristics was unknown before screening, *BRCA1/2* status was determined by central prospective germline testing during screening, and patients found to be germline *BRCA1/2* mutant were still enrolled. If possible, assessment of basal subtype was performed retrospectively.

Prior taxane and/or an anthracycline chemotherapy in the metastatic setting was allowed, alongside one other non-platinum-based chemotherapy in the first- or second-line (no restrictions were placed on prior immunotherapy or targeted treatment in the metastatic setting, unless combined with a cytotoxic agent).

Key exclusion criteria included: any prior platinum therapy in the metastatic setting (adjuvant or neoadjuvant platinum-based chemotherapy was permitted if this was completed within 6 months of screening); relapse within 3 months of completion of prior adjuvant or neoadjuvant chemotherapy; known *BRCA1/2* germline mutations, either determined and documented prior to screening or determined during screening; and documented intrinsic subtype other than basal by PAM50.

Full inclusion and exclusion criteria are provided in the supplementary information.

### Treatments

The patients enrolled in this cohort received the RP2D established in part B^25^; following enrollment, patients received berzosertib (140 mg/m^2^) on days 2 and 9, ~24 h after receiving cisplatin (75 mg/m^2^; day 1) in 21-day cycles.

A patient could receive berzosertib in combination with carboplatin if they had not progressed but were unable to tolerate treatment due to toxicities associated with cisplatin, or it was considered by the Investigator to be in their best interest. The starting doses following switching would be berzosertib (90 mg/m^2^) plus carboplatin (AUC 5 mg/min/mL).

Patients received treatment until PD, unacceptable toxicity, withdrawal, or non-compliance with study protocol.

### Objectives

The primary objectives of this study were to evaluate the safety, tolerability, and preliminary efficacy of berzosertib when combined with cisplatin in patients with advanced, basal, germline *BRCA1/2* wild-type TNBC, with or without *TP53* mutations. Exploratory objectives of this study included the evaluation of biomarkers potentially associated with response to berzosertib in combination with cisplatin.

### Assessments and endpoints

The primary safety endpoints were TEAEs; clinical laboratory values (chemistry, hematology, urinalysis, coagulation); ECG; and vital signs. TEAEs were defined as any AEs that were reported, or worsened, on or after study drug initiation, through the safety follow-up visit. Serious TEAEs were defined as any AEs that were a congenital or birth abnormality, resulted in persistent or significant disability or incapacity, required or prolonged in-patient hospitalization, were life-threatening or resulted in death, or were otherwise deemed medically important. Related TEAEs were defined as any AE reported by the Investigator to have a relationship to study treatment, or where the relationship was unknown.

All AEs were coded according to the Medical Dictionary for Regulatory Activities V21.0^43^ and graded according to the National Cancer Institute Common Terminology Criteria for Adverse Events v4.0^44^.

The primary efficacy endpoint of this study was the ORR, defined as the proportion of participants with a BOR of PR or CR (summarized as objective response [OR] according to RECIST 1.1), where both PR and CR were confirmed by repeat assessments performed no less than 4 weeks after the criteria for response was first met.

Tumor assessments were performed from baseline until either end of treatment or PD using RECIST 1.1 guidelines. Initial disease was documented using baseline imaging scans (computed tomography or magnetic resonance imaging) taken within 14 days prior to the first dose of study drug; imaging was repeated at the end of every 2 cycles for the first 12 cycles, followed by every 2 or 3 cycles, and finally 5 ± 1 weeks after completion of therapy.

The secondary efficacy endpoints of this study were PFS, DOR, OS, and clinical benefit rate (CBR). PFS was defined as the time from the date of first study drug dose to the first documentation of PD or death due to any cause, whichever occurred first. DOR was defined as the time that response criteria were first met for PR or CR until the date that recurrent or PD disease was objectively documented. OS was defined as the time from the date of the first dose of study drug to death due to any cause. CBR was defined as the proportion of patients who achieved a BOR of CR, PR, or SD of ≥6 months, measured from the date of first study drug dose.

Blood samples for PK analysis of berzosertib were collected pre-dose, 0.5 h before the end of infusion, at the end of infusion, and at 0.5, 1, 2, 3, and optionally at 7 h after the end of infusion on cycle 1, day 2. A limited berzosertib PK sampling scheme was implemented in this expansion cohort, with samples collected up to 4 h after the start of infusion for all participants and optionally at 8 h; therefore, only a partial AUC up to 4 h after the start of infusion (AUC_0–4_) was estimated for berzosertib. Berzosertib was administered by i.v. infusion over 60 (±10) min; if the total volume of infusion was ≥600 mL, infusion time could be extended up to 90 min, as tolerated. Tumor biopsies – either historical core or surgical specimens, or core biopsy obtained at screening (if the biopsy could be considered standard clinical practice) – were collected for potential exploratory evaluation of correlations between genetic alterations and treatment outcomes.

### Biomarker analyses

Germline BRCA1/2 mutational status was determined using the BRCAnalysis assay (Myriad Genetics, Inc., Salt Lake City, UT, USA) and tumor intrinsic subtype was assessed via PAM50 molecular profiling using the Breast Cancer Prognostic Gene Signature Assay (Prosigna®; LabCorp [Covance], Burlington, NC, USA).

Exploratory biomarkers – including LOH and TMB, surrogate markers for DNA repair deficiencies – were assessed using FMI’s FoundationOne® CDx next-generation sequencing (Foundation Medicine Inc., Cambridge, MA, USA). The genomic LOH score determined by FMI is assessed based on the percent of LOH in the tumor genome and is computed by inferring LOH regions across the 22 autosomal chromosomes using the genome-wide copy number profile and minor allele frequencies of the single-nucleotide polymorphisms. Regarding LOH, patients were categorized as having high or low LOH scores (≥16 or <16, respectively), while for TMB, patients were categorized (in somatic mutations per mega base [MB]) as high (≥20), intermediate (≥6–<20), or low (<6)^30,31^. The single nucleotide variants provided by the CDx assay were further analyzed for their functional consequences using the Variant Effect Predictor (VEP), Ensembl release 99® (Ensembl, Cambridge, UK)^45^. Using VEP annotation, single nucleotide and indel variations were grouped into the following categories, irrespective of the number of affected alleles: high impact, predicted high impact, and other. Only mutations with either a high or predicted high impact were considered in the analysis.

### Statistical analyses

Planned enrollment for this cohort was 50 patients, with a minimum of 30 patients who were basal subtype and *BRCA1/2* germline wild type; if *BRCA1/2* status was unavailable before screening it was to be determined prospectively at screening, if possible. Patients known to be non-basal subtype or *BRCA1/2* germline mutant were not screened for inclusion in this study.

Based on historical response rates of ~25% for single agent carboplatin^7,32^, the power for a one-sided method at different treatment response rates was calculated for a minimum of 30 patients who were basal subtype and *BRCA1/2* germline wild type. If at least 12 responses were observed (approximate ORR of 40%), the estimated 90% CI were calculated as 25.0% and 56.6%, respectively.

Four distinct analysis sets were established with the following definitions: SAF, all enrolled patients who received at least one dose of study drug; PAS, all enrolled patients who received at least one dose of berzosertib and provided at least one measurable post-dose concentration; mFAS, all enrolled patients who received at least one dose of study drug, had a baseline scan with a measurable target lesion; and mPES, all patients in the mFAS who were basal subtype and *BRCA1/2* germline wild type.

The primary efficacy endpoint of this study was analyzed in both the mFAS and the mPES. Each efficacy endpoint was calculated using two-sided 90% CI (OR and CBR using the Clopper-Pearson method^46^; PFS, OS, and DOR according to Brookmeyer and Crowley^47^).

Bioanalysis of berzosertib concentrations for PK analysis was performed in plasma samples using validated liquid chromatograph-tandem mass spectrometry methods in compliance with standard operating procedures. All PK analyses was conducted using standard non-compartmental analyses methods.

### Reporting summary

Further information on research design is available in the Nature Research Reporting Summary linked to this article.

## Supplementary information

SUPPLEMENTARY INFORMATION

Reporting Summary

## Data Availability

Any requests for data by qualified scientific and medical researchers for legitimate research purposes will be subject to the healthcare business of Merck KGaA, Darmstadt, Germany Data Sharing Policy. All requests should be submitted in writing to the healthcare business of he Merck KGaA, Darmstadt, Germany data sharing portal (https://www.merckgroup.com/en/research/our-approach-to-research-and-development/healthcare/clinicaltrials/commitment-responsible-data-sharing.html). When the healthcare business of Merck KGaA, Darmstadt, Germany has a co-research, co-development, co-marketing, or co-promotion agreement, or when the product has been out-licensed, the responsibility for disclosure might be dependent on the agreement between parties. Under these circumstances, the healthcare business of Merck KGaA, Darmstadt, Germany will endeavor to gain agreement to share data in response to requests.
