# Supplementary material for: Phase 1b study of berzosertib and cisplatin in patients with advanced triple-negative breast cancer
Source: NPJ Breast Cancer. 2022 Apr 7;8:45. doi: 10.1038/s41523-022-00406-0 (PMC8991212; doi:10.1038/s41523-022-00406-0)
Supplement: Supplementary file 1 — SUPPLEMENTARY INFORMATION [file 41523_2022_406_MOESM1_ESM.pdf]

SUPPLEMENTARY INFORMATION

Eligibility criteria[a]

Inclusion criteria

- Male and female participants ≥18 years of age.
- A male participant must agree to use and to have their female partners to use a highly effective contraception (i.e. methods with a failure rate of <1% per year) from the Screening visit, during the treatment period, and for at least 6 months after the last dose of study drug, and refrain from donating sperm during this period.
- A female participant is eligible to participate if she is not pregnant, not breastfeeding, and at least one of the following conditions applies: not a woman of childbearing potential (WOCBP) OR a WOCBP who agrees to use a highly effective contraception (i.e. methods with a failure rate of <1% per year) 28 days before start of first dose of study drug (as appropriate), during the treatment period, and for at least 6 months after the last dose of study drug.
- Advanced (locally advanced incurable or metastatic), histologically confirmed estrogen receptor, progesterone receptor, and human epidermal growth factor receptor 2 negative breast cancer.
- Adequate available historical tumor specimen (core biopsy or surgical specimen; fine needle aspirate inadequate) available for shipment to the Sponsor as confirmed by the Investigator unless otherwise agreed to by the Sponsor, or willing to provide a tumor biopsy (core) if the biopsy may be considered as part of standard clinical practice for the patient.
- Measurable disease according to Response Evaluation Criteria in Solid Tumors criteria (Version 1.1).
- World Health Organization performance status of 0 or 1.
- Life expectancy of ≥ 12 weeks.

- Hematological and biochemical indices within the following ranges at screening, with no clinically significant changes in these values confirmed on the first day of dosing, prior to study drug administration:
    - Hemoglobin ≥8.0 g/dL
    - Absolute neutrophil count ≥1.5 × 10^9/L
    - Platelet count ≥100 × 10^9/L
    - Serum bilirubin ≤1.5 × upper limit of normal (ULN) unless the participant has known or suspected Gilbert's syndrome
    - Alanine aminotransferase and aspartate transaminase ≤2.5 × ULN or ≤5 × ULN in the presence of liver metastases
    - Estimated glomerular filtration rate ≥50 mL/min
    - Prothrombin time <1.5 × ULN
    - No other clinically significant metabolic or hematologic abnormalities that were uncorrectable or that required ongoing, recurrent pharmacologic management.
- Signed and dated informed consent document.
- Willing and able to comply with scheduled visits, treatment plan, lifestyle, laboratory tests, contraceptive guidelines, and other study procedures.

Exclusion criteria

- Radiotherapy (except for palliative reasons), endocrine therapy, immunotherapy, or chemotherapy during the previous 4 weeks (6 weeks for nitrosoureas and Mitomycin-C, and 4 weeks for investigational medicinal products) or less than 4 drug half-lives, whichever is greater, before first dose of study drug.
- Prior chemotherapy.
- Any prior platinum therapy in the adjuvant, neoadjuvant, or metastatic setting within 6 months of screening.

- Relapse within 3 months of completion of prior adjuvant or neoadjuvant chemotherapy.
- Any prior chemotherapy in the metastatic setting with the exception of either a taxane and/or an anthracycline and one other non-platinum-based chemotherapy in the first- and second-line metastatic setting
    - There is no restriction on prior immunotherapy or targeted therapy in the metastatic setting unless combined with a cytotoxic agent.
- Participants with known *BRCA1/BRCA2* germline mutations, either determined and documented prior to Screening, or determined during Screening. Participants with unknown BRCA1/BRCA2 status may be enrolled at discretion of the Sponsor.
- Participants who are documented to be non-basal subtype using molecular profiling assay (e.g. Prediction Analysis of Microarray 50 [PAM50] assay) prior to Screening.
- Participants with unknown BRCA1/BRCA2 or basal subtype status will be enrolled until the number of enrolled participants is approximately 40. If approximately 40 participants have been enrolled and a minimum of 30 participants who are basal subtype positive and BRCA1/BRCA2 germline wild-type have not been enrolled, the basal subtype and BRCA status assay will be required at Screening to exclude participants who are basal subtype negative or have BRCA1/BRCA2 germline mutations.
- Unresolved toxicity of Common Terminology Criteria for Adverse Events (CTCAE) grade 2 or greater from previous anticancer therapy or radiotherapy, excluding:
    - Alopecia
    - Anemia or leukopenia, as long as screening hemoglobin and absolute neutrophil counts fall within limits specified in inclusion criteria
    - Other toxicities that, in the opinion of the Investigator and the Sponsor, should not exclude the participant.

- History of spinal cord compression or brain metastases, unless asymptomatic, treated, stable, and not requiring treatment with steroids for at least 4 weeks before first dose of study drug. Any history of leptomeningeal metastases.
- Female participants who are already pregnant or lactating, or plan to become pregnant within 6 months of the last dose of study drug are excluded. Female participants of childbearing potential must adhere to contraception guidelines. Female participants will be considered non-childbearing potential if they have undergone surgical hysterectomy or bilateral oophorectomy or have been amenorrheic for over 2 years with a screening serum follicle stimulating hormone level within the laboratory’s reference range for postmenopausal females.
- Male participants with partners of child-bearing potential must agree to adhere to contraception guidelines. Men with pregnant or lactating partners or partners who plan to become pregnant during the study or within 6 months of the last dose of study drug are excluded.
- Major surgery ≤2 weeks before starting study drug, or incomplete recovery from a prior major surgical procedure.
- Cardiac conditions as follows:
    - Clinically significant cardiovascular event within 6 months before study entry to include:
        - congestive heart failure requiring therapy
        - unstable angina pectoris
        - myocardial infarction
        - Class II/III/IV cardiac disease (New York Heart Association)
        - presence of severe valvular heart disease
        - presence of a ventricular arrhythmia requiring treatment.

    - History of arrhythmia that is symptomatic or requires treatment (CTCAE 3), symptomatic or uncontrolled atrial fibrillation, despite treatment, or asymptomatic sustained ventricular tachycardia. Participants with atrial fibrillation controlled by medication are permitted
    - Uncontrolled hypertension (blood pressure ≥160/100 mmHg despite optimal therapy).
    - Second- or third-degree heart block with or without symptoms
    - QTc >450 msec (by Fridericia's correction) not due to electrolyte abnormality and that does not resolve with correction of electrolytes
    - History of congenital long QT syndrome
    - History of torsades de pointes (or any concurrent medication with a known risk of inducing torsades de pointes)
    - Clinically significant abnormality, including ejection fraction below normal institutional limits, present on transthoracic echocardiogram performed at Screening.
- Prior bone marrow transplant or extensive radiotherapy to greater than 15% of bone marrow.
- Participation or plan of participation in another interventional clinical study while taking part in this phase 1 study of M6620. Participation in an observational study would be acceptable.
- Any other condition that, in the Investigator's opinion, would not make the participant a good candidate for the clinical study, including:
    - History of HIV-1, HIV-2, hepatitis C virus, or unresolved hepatitis B infection
    - High medical risk because of non-malignant systemic disease. including active uncontrolled infection
    - History of serious drug allergy or auto-immune disease

    - Participants who have been diagnosed with Li-Fraumeni Syndrome or ataxia telangiectasia.
- Current malignancies other than triple negative breast cancer (TNBC) (C2), with the exception of adequately treated cone-biopsied in situ carcinoma of the cervix uteri and basal or squamous cell carcinoma of the skin. Participants with prior non-TNBC malignancies who have been in remission for less than 3 years are excluded.
- Participant is the Investigator or a Sub-Investigator, Research Assistant, Pharmacist, Study Coordinator, other staff, or a relative of study personnel directly involved with the conduct of the study.
- Participants receiving treatment with medications that are known to be strong inhibitors or inducers of cytochrome P450 (CYP) 3A4 that cannot be discontinued at least a week before start of treatment and for the duration of the study.
- Participants receiving treatment with ototoxic or nephrotoxic medications that cannot be discontinued at least 7 days before first dose of study drug and for the duration of the study. Inadvertent or short-term use on study will not cause a participant to be ineligible. If a short course of therapy with nephrotoxic or ototoxic medication is anticipated and required, cisplatin and carboplatin should be discontinued until 7 days after this course is completed.

[a]The eligibility criteria listed here are written as presented in version 15.0 of the protocol (Supplementary Materials and Methods, redacted protocol, pages 87–93)

Supplementary Figure 1. Berzosertib plasma concentration-time profiles after the first intravenous infusion for berzosertib lead-in period (140 mg/m^2) and berzosertib (140 mg/m^2) + cisplatin (40–75 mg/m^2) cohorts (Part B) and berzosertib (140 mg/m^2) + cisplatin (75 mg/m^2) cohort (Part C2).

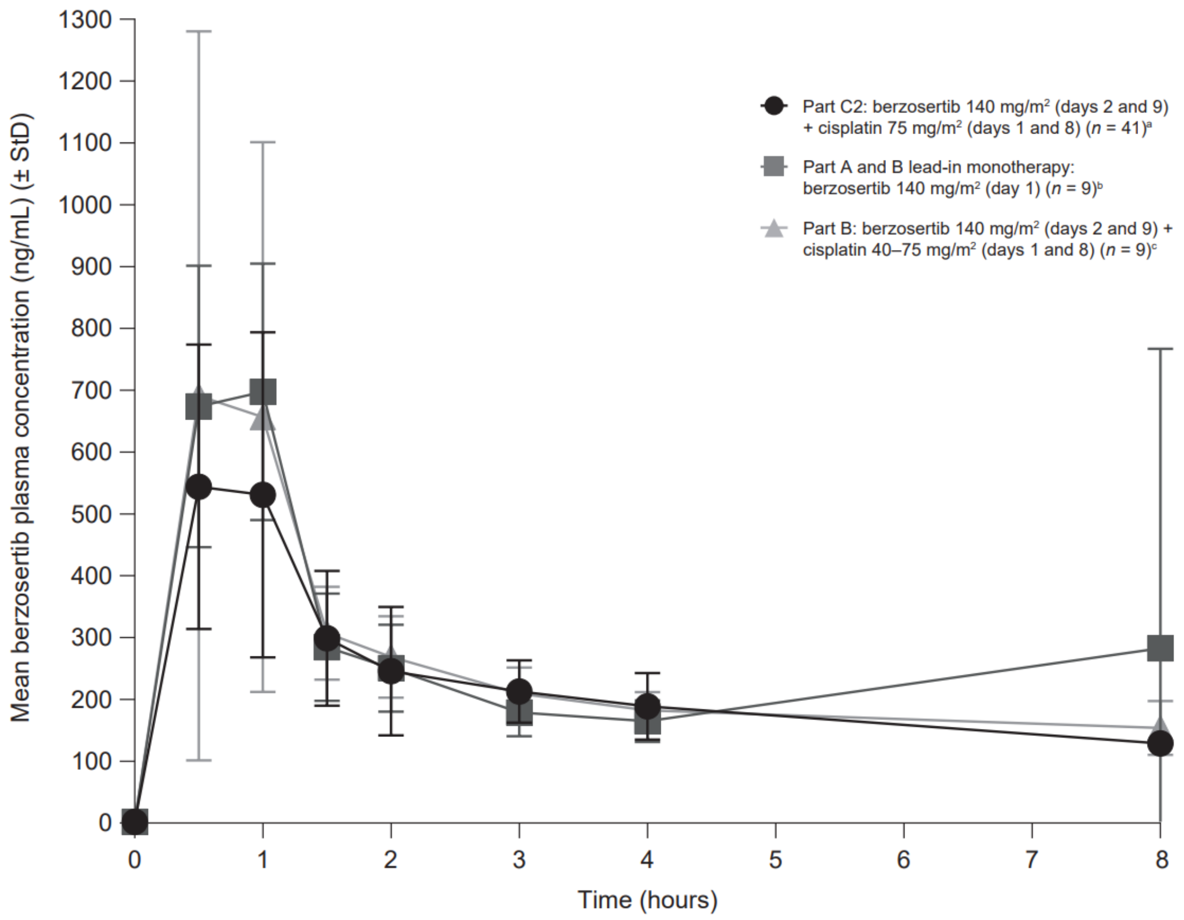

[a]In the part C2 group: *N* = 40 before infusion, *N* = 27 for 0.5-hour timepoint, *N* = 22 for 1-hour timepoint, *N* = 37 for 1.5-hour timepoint, *N* = 39 for 2-hour timepoint, *N* = 36 for 3-hour timepoint, *N* = 40 for 4-hour timepoint, *N* = 2 for 8-hour timepoint. [b]In the part A and B lead-in monotherapy group: *N* = 8 for 1.5-hour timepoint, *N* = 4 for 3-hour timepoint; [c]In the Part B berzosertib + cisplatin group: *N* = 8 for 2-, 4-, and 8-hour timepoints, *N* = 7 for 3-hour timepoint.

StD, standard deviation.

Supplementary Table 1. Best overall response to treatment by number of prior lines of therapy for metastatic disease.

Efficacy outcome	Prior lines of therapy for metastatic disease<br>Patients, *N* (%) unless stated			
Best overall response	Neoadjuvant/adjuvant	1st line for metastatic disease	2nd line for metastatic disease	>2nd line for metastatic disease
CR	2	0	0	0
PR	6	2	1	0
SD	9	6	1	0
PD	4	3	4	3
Not evaluable	0	3	0	1

CR, complete response; PD, progressive disease; PR, partial response; SD, stable disease.

Supplementary Table 2. PFS time for selected biomarker subgroups; modified full analysis set (*N* = 47).

Gene	Patients, n	Events, n	Median PFS, months (90% CI)
TP53[a]			
Wild type	2	2	3.0 (2.6, 3.4)
Mutant	34	28	4.1 (2.8, 6.0)
ARID1A			
Wild type	34	282	4.1 (3.1, 6.0)
Mutant	2		1.9 (1.2, 2.6)
ATM			
Wild type	31	26	4.1 (2.8, 6.0)
Mutant	5	4	2.6 (1.2, nd)
Germline BRCA1/2			
Wild type	31	27	3.4 (2.6, 5.8)
Mutant	5	3	6.9 (1.6, nd)
Basal subtype[b]			
Yes	36	26	4.1 (2.8, 6.2)
No	7	7	3.4 (1.2, 6.9)

[a]Only patients with biomarker status determined by FoundationOne® CDx next generation sequencing were reported. [b]basal subtype was determined via PAM50 analysis (Prosigna).

ARID1A, AT-rich interaction domain 1A; *ATM,* ataxia-telangiectasia mutated; *BRCA1/2,* breast cancer gene 1/2; CI, confidence interval; nd, not defined; PAM50, Prediction Analysis of Microarray 50; PFS, progression-free survival; *TP53,* tumor protein 53.

Supplementary Table 3. Statistical analysis for the association of high vs low LOH with clinical response (Fisher's Exact Test)

BOR	High LOH	Low LOH	Unknown	No data	Sum
CR	-	1	-	1	2
PR	7	-	1	1	9
SD	4	5	6	3	18
PD	4	6	1	3	14
NE	1	-	-	3	4
Sum	16	12	8	11	47

The statistical test compares only the objective responses in LOH high versus LOH low tumors colored in gray:

Fisher's Exact Test for Count Data

p-value = 0.088

alternative hypothesis: true odds ratio is not equal to 1

95 percent confidence interval: 0.7823737 418.7744010

sample estimates: **odds ratio 7.96**

The test is not significant at a significance level of 0.05 but shows enrichment of responders in LOH high tumors (OR=7.96).

BOR, best overall response; CR, complete response; LOH, loss of heterozygosity; PD, progressive disease; PR, partial response; SD, stable disease

Supplementary Table 4. ATM mutations and allelic status

Patient ID	Response	Gene	Protein	CDS	Position	Depth	AF	Zygosity	Impact (VEP)
1	PR	ATM	E166K	496G>A	chr11:108106561	776	6.31	NA	Moderate
2	PD	ATM	D1790N	5368G>A	chr11:108173628	724	28.18	NA	Moderate
3	SD	ATM	G204R	610G>A	chr11:108114793	719	45.48	Heterozygous	Moderate
4	PD	ATM	E2187*	6559G>T	chr11:108192134	634	34.38	Heterozygous	High
5	PD	ATM	N1230S	3689A>G	chr11:108153549	747	77.11	Homozygous	Moderate

AF, Allele frequency; ATM, ataxia-telangiectasia mutated; CDS, coding DNA sequence; PD, progressive disease; PR, partial response; SD, stable disease; VEP, Variant effect predictor

Supplementary Table 5. Individual biomarker data by response.

Patient ID	Gene	CDS	Protein	Position	Depth	AF	Response
6	*ARID1A*	2718C>G	N906K	chr1:27089762	1020	49.12	PD
7	*ARID1A*	4001_4002insGCA	Q1334_R1335insQ	chr1:27100205	1221	26.86	SD
1	*ATM*	496G>A	E166K	chr11:108106561	776	6.31	PR
2	*ATM*	5368G>A	D1790N	chr11:108173628	724	28.18	PD
3	*ATM*	610G>A	G204R	chr11:108114793	719	45.48	SD
4	*ATM*	6559G>T	E2187*	chr11:108192134	634	34.38	PD
5	*ATM*	3689A>G	N1230S	chr11:108153549	747	77.11	PD
8	*BRCA2*	7933A>G	R2645G	chr13:32936787	1137	60.07	SD
9	*BRCA2*	5897A>G	H1966R	chr13:32914389	527	45.92	SD
10	*BRCA2*	7250A>C	H2417P	chr13:32929240	930	5.38	PR

11	*BRCA2*	8940_8941insA	E2981fs*37	chr13:32953639	270	56	PR
12	*BRCA2*	9976A>T	K3326*	chr13:32972626	1116	60.3	PD
13	*MDM4*	391C>T	P131S	chr1:204506605	469	23.88	PR
1	*TP53*	660T>A	Y220*	chr17:7578189	751	84.55	PR
14	*TP53*	584T>C	I195T	chr17:7578265	947	41.39	PD
15	*TP53*	917_919+6delGAGGTAAGC	splice site 917_919+6delGAGGTAAGC	chr17:7577012	1058	32.14	SD
16	*TP53*	778delT	S260fs*85	chr17:7577502	672	41.67	PD
13	*TP53*	329_330insGTTTCCG	L111fs*40	chr17:7579357	477	22.64	PR
17	*TP53*	329G>T	R110L	chr17:7579358	648	18.36	SD
2	*TP53*	220delG	A74fs*49	chr17:7579466	906	36.98	PD
18	*TP53*	74+5_83del122	splice site 74+5_83del122	chr17:7579712	1144	49.3	SD

3	*TP53*	438G>A	W146*	chr17:7578492	628	32.96	SD
6	*TP53*	637C>T	R213*	chr17:7578212	1166	37.65	PD
19	*TP53*	818G>A	R273H	chr17:7577120	875	90.06	*NA*
8	*TP53*	488A>G	Y163C	chr17:7578442	939	46.43	SD
21	*TP53*	524G>A	R175H	chr17:7578406	888	43.36	CR
22	*TP53*	715A>G	N239D	chr17:7577566	633	70.77	PD
23	*TP53*	586C>T	R196*	chr17:7578263	304	52.3	PD
24	*TP53*	321C>A	Y107*	chr17:7579366	1078	30.06	SD
25	*TP53*	582_583insC	I195fs*14	chr17:7578266	487	73.31	PR
4	*TP53*	1024C>T	R342*	chr17:7574003	713	32.12	PD
26	*TP53*	818G>A	R273H	chr17:7577120	1148	68.03	SD
27	*TP53*	527G>T	C176F	chr17:7578403	570	62.28	SD

9	*TP53*	606_638del33	V203_R213del	chr17:7578210	440	5.91	SD
28	*TP53*	524G>A	R175H	chr17:7578406	1013	57.95	SD
29	*TP53*	404G>A	C135Y	chr17:7578526	559	63.86	PR
10	*TP53*	832C>T	P278S	chr17:7577106	1071	40.62	PR
30	*TP53*	113_138del26	Q38fs*5	chr17:7579548	464	59.05	PR
11	*TP53*	537T>A	H179Q	chr17:7578393	336	66	PR
31	*TP53*	809T>G	F270C	chr17:7577129	558	57.71	SD
5	*TP53*	733G>T	G245C	chr17:7577548	783	62.07	PD
32	*TP53*	395A>G	K132R	chr17:7578535	634	61.67	PD
12	*TP53*	853G>A	E285K	chr17:7577085	987	23.4	PD
33	*TP53*	746G>C	R249T	chr17:7577535	800	5.25	SD
34	*TP53*	947_964CCCAGCCA AAGAAGAAAC>T	P316fs*15	chr17:7576882	1179	6.62	PR

35	*TP53*	1024C>T	R342*	chr17:7574003	770	6.88	SD

AF, Allele frequency; *ARID1A,* AT-rich interaction domain 1A; *ATM*, ataxia-telangiectasia mutated; *BRCA1/2,* breast cancer gene 1/2; CDS, coding DNA sequence; PD, progressive disease; PR, partial response; SD, stable disease; *TP53,* tumor protein 53; VEP, Variant effect predictor
