# Supplementary material for: Phase 1b study of berzosertib and cisplatin in patients with advanced triple-negative breast cancer
Source: NPJ Breast Cancer. 2022 Apr 7;8:45. doi: 10.1038/s41523-022-00406-0 (PMC8991212; doi:10.1038/s41523-022-00406-0)
Supplement: Supplementary file 2 — Reporting Summary [file 41523_2022_406_MOESM2_ESM.pdf]

nature portfolio

Corresponding author(s): Ruth Plummer

Last updated by author(s): Dec 10, 2020

Reporting Summary

Nature Portfolio wishes to improve the reproducibility of the work that we publish. This form provides structure for consistency and transparency in reporting. For further information on Nature Portfolio policies, see our Editorial Policies and the Editorial Policy Checklist.

Statistics

For all statistical analyses, confirm that the following items are present in the figure legend, table legend, main text, or Methods section.

n/a	Confirmed	
☐	☒	The exact sample size (n) for each experimental group/condition, given as a discrete number and unit of measurement
☐	☒	A statement on whether measurements were taken from distinct samples or whether the same sample was measured repeatedly
☐	☒	The statistical test(s) used AND whether they are one- or two-sided<br>*Only common tests should be described solely by name; describe more complex techniques in the Methods section.*
☐	☒	A description of all covariates tested
☐	☒	A description of any assumptions or corrections, such as tests of normality and adjustment for multiple comparisons
☐	☒	A full description of the statistical parameters including central tendency (e.g. means) or other basic estimates (e.g. regression coefficient) AND variation (e.g. standard deviation) or associated estimates of uncertainty (e.g. confidence intervals)
☐	☒	For null hypothesis testing, the test statistic (e.g. F, t, r) with confidence intervals, effect sizes, degrees of freedom and P value noted<br>*Give P values as exact values whenever suitable.*
☒	☐	For Bayesian analysis, information on the choice of priors and Markov chain Monte Carlo settings
☐	☒	For hierarchical and complex designs, identification of the appropriate level for tests and full reporting of outcomes
☐	☒	Estimates of effect sizes (e.g. Cohen's d, Pearson's r), indicating how they were calculated

Our web collection on statistics for biologists contains articles on many of the points above.

Software and code

Policy information about availability of computer code

Data collection	*Provide a description of all commercial, open source and custom code used to collect the data in this study, specifying the version used OR state that no software was used.*
Data analysis	FMI's FoundationOne® CDx next generation sequencing (Foundation Medicine Inc., Cambridge, MA, USA); Variant Effect Predictor (VEP), Ensembl release 99® (Ensembl, Cambridge, UK)

For manuscripts utilizing custom algorithms or software that are central to the research but not yet described in published literature, software must be made available to editors and reviewers. We strongly encourage code deposition in a community repository (e.g. GitHub). See the Nature Portfolio guidelines for submitting code & software for further information.

Data

Policy information about availability of data

All manuscripts must include a data availability statement. This statement should provide the following information, where applicable:

- Accession codes, unique identifiers, or web links for publicly available datasets
- A description of any restrictions on data availability
- For clinical datasets or third party data, please ensure that the statement adheres to our policy

Any requests for data by qualified scientific and medical researchers for legitimate research purposes will be subject to the healthcare business of Merck KGaA, Darmstadt, Germany Data Sharing Policy. All requests should be submitted in writing to the healthcare business of he Merck KGaA, Darmstadt, Germany data sharing portal (https://www.merckgroup.com/en/research/our-approach-to-research-and-development/healthcare/clinicaltrials/commitment-responsible-data-sharing.html).
When the healthcare business of Merck KGaA, Darmstadt, Germany has a co-research, co-development, co-marketing, or co-promotion agreement, or when the

product has been out-licensed, the responsibility for disclosure might be dependent on the agreement between parties. Under these circumstances, the healthcare business of Merck KGaA, Darmstadt, Germany will endeavor to gain agreement to share data in response to requests.

Field-specific reporting

Please select the one below that is the best fit for your research. If you are not sure, read the appropriate sections before making your selection.

☒ Life sciences ☐ Behavioural & social sciences ☐ Ecological, evolutionary & environmental sciences

For a reference copy of the document with all sections, see nature.com/documents/nr-reporting-summary-flat.pdf

Life sciences study design

All studies must disclose on these points even when the disclosure is negative.

Sample size	Based on historical response rates of approximately 25% for single agent carboplatin7,32, the power for a one-sided method at different treatment response rates was calculated for a minimum of 30 patients who were basal subtype and BRCA1/2 germline wild type. If at least 12 responses were observed (approximate ORR of 40%), the estimated 90% CI were calculated as 25.0% and 56.6%, respectively.
Data exclusions	Key exclusion criteria included: any prior platinum therapy in the metastatic setting (adjuvant or neoadjuvant platinum-based chemotherapy was permitted if this was completed within 6 months of screening); relapse within 3 months of completion of prior adjuvant or neoadjuvant chemotherapy; known BRCA1/2 germline mutations, either determined and documented prior to screening or determined during screening; and documented intrinsic subtype other than basal by PAM50.<br>Full inclusion and exclusion criteria are provided in the supplementary information.
Replication	The primary efficacy endpoint of this study was analyzed in both the mFAS and the mPES. Each efficacy endpoint was calculated using 2-sided 90% CI (OR and CBR using the Clopper-Pearson method46; PFS, OS and DOR according to Brookmeyer and Crowley47).<br>Bioanalysis of berzosertib concentrations for PK analysis was performed in plasma samples using validated liquid chromatograph-tandem mass spectrometry methods in compliance with standard operating procedures. All PK analyses was conducted using standard non-compartmental analyses methods.
Randomization	Part C2 was a single-arm, dose expansion cohort evaluating the safety and preliminary efficacy of berzosertib combined with cisplatin in patients with advanced TNBC using the berzosertib RP2D
Blinding	This was an open-label study

Reporting for specific materials, systems and methods

We require information from authors about some types of materials, experimental systems and methods used in many studies. Here, indicate whether each material, system or method listed is relevant to your study. If you are not sure if a list item applies to your research, read the appropriate section before selecting a response.

Materials & experimental systems

n/a	Involved in the study	
☒	☐	Antibodies
☒	☐	Eukaryotic cell lines
☒	☐	Palaeontology and archaeology
☒	☐	Animals and other organisms
☐	☒	Human research participants
☐	☒	Clinical data
☒	☐	Dual use research of concern

Methods

n/a	Involved in the study	
☒	☐	ChIP-seq
☒	☐	Flow cytometry
☒	☐	MRI-based neuroimaging

Human research participants

Policy information about studies involving human research participants

Population characteristics	Forty-seven patients were enrolled into this study, all of whom were included in the safety analysis set (SAF) and modified full analysis set (mFAS) for efficacy (Table 1). The PK analysis set (PAS) included all enrolled patients who received at least one dose of berzosertib and provided at least one measurable post-dose concentration; 41 patients were included in the PAS, with six patients excluded as they did not have a measurable post-dose PK berzosertib concentration. The modified primary efficacy set (mPES) included all patients in the mFAS who were basal subtype and BRCA1/2 germline wild-type; 35 patients were included in the mPES, with 12 patients excluded due to a lack of BRCA1/2 gWT status data or were not basal subtype. Among all patients, 34 (72.3%) were TP53 mutant; two (4.3%) were TP53 wild type; 31 (66%) were BRCA1/2 germline wild-type; and 36 (76.6%) were basal subtype, based on Prediction Analysis of Microarray 50 (PAM50). In the seven patients who were not basal subtype, four (57.1%) had a TP53 mutation, one (14.3%) was TP53 wild-type and two (28.6%) had an unknown TP53 mutational status; in the 36 (76.6%) patients who were basal subtype, 29 (81%) had a TP53 mutation, one (3%) was

	TP53 wild-type and six (17%) had an unknown TP53 mutational status. Four patients had an unknown subtype (Table 2). Twenty-four (51.1%) patients had previously received only one line of therapy for metastatic disease, 10 (21.3%) patients had received two lines of therapy, and five (10.6%) received more than two lines of therapy for metastatic disease.
Recruitment	Participants enrolled were selected based on biomarker status. Within the TNBC population, the basaloid subtype, which exhibits even higher rates of TP53 mutation (approximately 85%; Cosmic Database), derives modest benefit from platinum therapy. Thus, this subtype has the potential to achieve added benefit with the addition of berzosertib.TNBC patients with BRCA1/2 germline mutations appear to derive the most benefit from single-agent platinum, supporting the notion that defects in homologous recombination drive sensitivity to platinum. In view of the potential challenge of demonstrating any additional benefit of berzosertib in this subpopulation (Tutt 2014), participants with basaloid positive, BRCA1/2 gWT TNBC will primarily be selected for the evaluation of safety and efficacy of berzosertib in combination with cisplatin in Part C2.
Ethics oversight	Prior to initiation of the study at a given site, the Clinical Study Protocol (CSP) and all required associated documents, including any information presented to potential study participants were approved by the responsible Institutional Review Board (IRB) or Independent Ethics Committee (IEC). Subsequent protocol amendments were also submitted to the responsible IEC or IRB, before implementation of substantial changes.<br><br>The study was conducted in accordance with the ethical principles of the International Council for Harmonisation (ICH) guideline for Good Clinical Practice (GCP) and the Declaration of Helsinki, as well as with applicable local regulations.

Note that full information on the approval of the study protocol must also be provided in the manuscript.

Clinical data

Policy information about clinical studies

All manuscripts should comply with the ICMJE guidelines for publication of clinical research and a completed CONSORT checklist must be included with all submissions.

Clinical trial registration	NCT02157792
Study protocol	The redacted protocol has been provided
Data collection	Patients were enrolled across five sites in the UK and 12 in the USA, between December 2015 (study initiation date) and March 2020 (study completion date, when the final patient completed their last visit).
Outcomes	The primary safety endpoints were TEAEs; clinical laboratory values (chemistry, hematology, urinalysis, coagulation); ECG; and vital signs. TEAEs were defined as any AEs that were reported, or worsened, on or after study drug initiation, through the safety follow-up visit. Serious TEAEs were defined as any AEs that were a congenital or birth abnormality, resulted in persistent or significant disability or incapacity, required or prolonged in-patient hospitalization, were life-threatening or resulted in death, or were otherwise deemed medically important. Related TEAEs were defined as any AE reported by the Investigator to have a relationship to study treatment, or where the relationship was unknown.<br>All AEs were coded according to the Medical Dictionary for Regulatory Activities V21.0 and graded according to the National Cancer Institute Common Terminology Criteria for Adverse Events v4.0.<br>The primary efficacy endpoint of this study was the ORR, defined as the proportion of participants with a BOR of PR or CR (summarized as objective response [OR] according to RECIST 1.1), where both PR and CR were confirmed by repeat assessments performed no less than 4 weeks after the criteria for response was first met.<br>Tumor assessments were performed from baseline until either end of treatment or PD using RECIST 1.1 guidelines. Initial disease was documented using baseline imaging scans (computed tomography or magnetic resonance imaging) taken within 14 days prior to the first dose of study drug; imaging was repeated at the end of every 2 cycles for the first 12 cycles, followed by every 2 or 3 cycles, and finally 5±1 weeks after completion of therapy.<br>The secondary efficacy endpoints of this study were PFS, DOR, OS, and clinical benefit rate (CBR). PFS was defined as the time from the date of first study drug dose to the first documentation of PD or death due to any cause, whichever occurred first. DOR was defined as the time that response criteria were first met for PR or CR until the date that recurrent or PD disease was objectively documented. OS was defined as the time from the date of the first dose of study drug to death due to any cause. CBR was defined as the proportion of patients who achieved a BOR of CR, PR, or SD of ≥6 months, measured from the date of first study drug dose.
